# Editorial: Insight in pediatric pain – 2023

**DOI:** 10.3389/fpain.2024.1437873

**Published:** 2024-06-21

**Authors:** Anthony Herbert, Michael P. Jankowski

**Affiliations:** ^1^ Padiatric Palliative Care Service, Children’s Health Queensland Hospital and Health Service, Brisbane, QLD, Australia; ^2^ Centre for Children’s Health Research, Queensland University of Technology, Brisbane, QLD, Australia; ^3^ Department of Anesthesia, Division of Pain Management, Cincinnati Children’s Hospital Medical Center, Cincinnati, OH, United States; ^4^ Pediatric Pain Research Center, Cincinnati Children’s Hospital Medical Center, Cincinnati, OH, United States

**Keywords:** pediatrics, nociception, guidelines, neonatal, pain, development

**Editorial on the Research Topic** Insight in pediatric pain – 2023

The International Association for the Study of Pain (IASP) defines pain as “an unpleasant sensory and emotional experience associated with, or resembling that associated with, actual or potential tissue damage” (1). Pain can be experienced at all ages across the lifespan, but it is known that mechanisms by which pain is produced early in life are distinct from that observed in adolescents or adults [e.g., (2–4)]. It is therefore crucial to study pain processing across development at the basic, translational and clinical levels to develop novel therapies for pain in children.

This research Topic was designed to bring together new information on Pediatric Pain from basic science and clinical researchers to discuss innovative ideas in understudied areas of pain research. The first article by Ullsten et al. entitled, “*Parent-led neonatal pain management –a narrative review and update of research and practices*” discusses how parents are becoming a key player in appropriate pain management for neonates. Relatively simple tasks such as skin-to-skin contact, breastfeeding and parent vocalizations are empowering parents to assess and manage their child's pain. These practices are especially important for neonates that experience repeated procedures in the hospital setting as research has proven these methods to be excellent adjuncts to standards of care. The study raises an interesting question of whether some of these non-standard methods can be used by older patients for pain management.

This is a perfect setup to our next study by Wallbing et al. entitled, “*Help Overcoming Pain Early (HOPE), a brief person-centred intervention for adolescents with chronic pain in a school setting, may improve symptoms of insomnia*”. It is well known that patients with chronic pain display sleep disturbances. This is especially true for adolescent patients. This report analyzed the impact of the HOPE intervention on insomnia and self-related health in adolescents. The report shows statistically significant improvements in sleep and self-related health at a six month follow up in adolescents with chronic pain. Results again support the notion that small interventions such as HOPE can not only provide pain management, but also improve co-morbidities associated with chronic pain in adolescents. The study also highlights the importance of how pain can influence school performance, physical activities, socialisation and other hobbies. There is certainly a role for medications in pain management, but non-pharmacological strategies including physiotherapy and psychological therapies should always be considered.

Finally, many providers have legitimate concerns about using opioids in chronic pain patients. Ample data suggests that long-term use of opioids and opioid consumption in chronic pain has limited benefits that may outweigh any analgesia. In the report by Taylor et al. entitled, “*Body Size and Brain Volumetry in the Rat Following Prolonged Morphine Administration in Infancy and Adulthood*” the authors show that prolonged exposure of rodents to opioids during the neonatal period altered body weight and brain volume. Although these effects appeared transient, subsequent dosing of opioids in adulthood of neonatally exposed rodents further increased susceptibility to altered brain volume. It is therefore important to consider the duration of opioid usage during infancy and how this could influence overall health in the long term.

Nevertheless, there could still be a role for prolonged opioids in patients with rare diseases comorbid with chronic pain or those receiving end of life care. For example, Cao et al. discuss in their article, “*Chronic Pain in a Pediatric Patient with Late-Onset Pompe Disease*”, that pain may have been further influenced by the patient's depressed mood, anxiety and attention related concerns. In this case, clinicians recommend a multidisciplinary treatment approach that could include pharmacotherapies.

The idea of interdisciplinary pain management is supported by guidelines developed by the World Health Organization (WHO). “Cancer Pain Relief and Palliative Care in Children” was a groundbreaking publication of the IASP and WHO published in 1998 (5). It emphasized the clinical necessity of timely pain management for children using a Pain Management ladder. Further, it emphasized the broader context of suffering beyond physical pain, the need for both comprehensive assessment and management of pain as well as non-pharmacological strategies to manage pain.

The most recent guideline “Guidelines for the Management of Chronic Pain in Children” was published in 2020. There has been a move away from the “ladder” to using a stepwise approach. These guidelines also emphasize the ethical imperative to manage pain in children with both cancer and non-cancer diagnoses. The Guideline appropriately emphasizes the Biopsychosocial perspective in managing pain. It acknowledges that there are significant gaps in research which in part was the motivation for this special edition on research in paediatric pain. The guideline acknowledges the strong evidence for the role of physical therapies, especially in the management of chronic primary pain conditions (particularly musculoskeletal pain). There is also good evidence of psychological strategies or mind-body approaches to managing pain as highlighted in the manuscript by Wallbing et al.

The role of opioids in managing chronic pain in children remains in evolution. Current recommendations are that there is currently not a role for opioids for those with chronic primary pain conditions (e.g., chronic daily headache, recurrent abdominal pain and other musculoskeletal conditions). However, the WHO Guidelines (6) do state appropriate pharmacological management tailored to specific indications may include the use of morphine for end-of-life-care. Further, the guidelines also suggest that in children with chronic pain associated with life-limiting conditions, morphine may be given by appropriately trained healthcare providers, under the principles of opioid stewardship.

In conclusion, with the complexity of managing pain in children, it is important to consider multiple avenues including pharmacotherapies, psychological therapy including cognitive behavioral therapy and physical therapy. In addition, it is important to engage parents in this process and approach pain management in children from a biopsychosocial-spiritual^ perspective. The integrated and interprofessional team approach should be tailored to the individual as best as possible and consider items such as those proposed in the articles of this collection. Hopefully in the future with more research, there will be better ways to manage chronic pain in children.
